# Correlating Ultrasonographic Features of Lymph Nodes During Endobronchial Ultrasound With Final Outcome

**DOI:** 10.7759/cureus.26554

**Published:** 2022-07-04

**Authors:** Tinku Joseph, Satish Reddy, Nitesh Gupta, Arvind Perathur, Archana George, Vidhya Chandraprabha, Namitha Shajil

**Affiliations:** 1 Pulmonary Medicine, Amrita Hospital, Kochi, IND; 2 Pulmonary, Critical Care and Sleep Medicine, Vardhman Mahavir Medical College and Safdarjung Hospital, Delhi, IND; 3 Pathology, Amrita Hospital, Kochi, IND

**Keywords:** lung cancer, mediastinum, ebus tbna, endobronchial ultrasound guided fine needle aspiration, mediastinal lymphadenopathy

## Abstract

Background

In clinical practice, metastatic primary lung cancer, TB, and sarcoidosis are the commonest causes of mediastinal lymphadenopathy. Differentiation of malignant from benign causes is essential. The sonographic features seem to correlate with malignancy in head and neck, breast, and cervix cancers and can be used to predict the etiology of lesions. The objective of our study was to assess the utility of different ultrasonographic features in differentiating benign and malignant lymph nodes by endobronchial ultrasound-guided transbronchial needle aspiration (EBUS-TBNA).

Methods

This is a prospective study analysis of all patients above 18 years presenting with mediastinal and hilar adenopathy on computed chest tomography with contrast, undergoing EBUS-TBNA for diagnosis. Lymph node ultrasonographic characteristics such as size, shape, echogenicity, margins, central hilar structure (CHS), and coagulation necrosis sign (CNS) were recorded and compared with histopathology.

Results

A total of 576 patients underwent the EBUS-TBNA procedure, and a total of 810 lymph nodes were evaluated. Three hundred and forty-eight patients (468 lymph nodes) were malignant; 228 patients (342 lymph nodes) were benign. Heterogeneous echotexture was significantly higher in malignant lymph nodes (<0.001). The multivariate analysis revealed that heterogeneous echotexture was an independent predictor for malignant etiology.

Conclusion

Heterogeneous echotexture of the lymph node on EBUS was predictive of malignancy. If heterogenicity is observed on EBUS, subsequent sampling of that lymph node might be considered, which may yield a higher diagnostic yield and may reduce the number of lymph nodes requiring sampling and further invasive procedures.

## Introduction

In routine day-to-day clinical practice, metastatic primary lung cancer, TB, and sarcoidosis are the commonest causes of mediastinal lymphadenopathy. Histopathological diagnosis of tissue is necessary for the management of mediastinal lymphadenopathy. Endoscopic ultrasound-guided fine-needle aspiration (EUS-FNA) and endobronchial ultrasound-guided transbronchial needle aspiration (EBUS-TBNA) are non-surgical techniques for the sampling and diagnosis of mediastinal lymphadenopathy. Convex probe endobronchial ultrasound allows characterisation of the lymph node. The round shape, distinct margin, heterogenous echogenicity, absence of central hilar structure (CHS), and the presence of coagulation necrosis sign (CNS) may help to differentiate benign from malignant lymph nodes [1-9]. Identification of lymph nodal characteristics on EBUS is especially helpful in decision-making in the background of non-diagnostic EBUS. This will assist in identifying the target lymph nodes with the highest pre-test probability, thus reducing the number of lymph nodes requiring sampling, especially in resource-limited settings. In the present study, we prospectively evaluated the ultrasonographic features of lymph nodes during EBUS and compared them with the final outcome, whether benign or malignant.

## Materials and methods

Selection and description of participants

The present study was a prospective observational study, performed in a tertiary care center over a period of four years (2017-2021). Consecutive patients, above 18 years of age, were enrolled. The study has been approved by the institutional review board. Informed consent was taken from all patients before the procedure.

Patients who presented to the pulmonary medicine department with mediastinal lymphadenopathy and had indications for EBUS were enrolled in the study. The exclusion criteria were severe hypoxemia, uncorrectable coagulopathy, life-threatening cardiac arrhythmias, recent myocardial infarction, severe pulmonary hypertension, and uremia.

Study protocol

Patients were inquired about demographic details (age, sex), clinical history (symptoms, duration, and comorbidities), and radiology (lymph node stations) and were documented in a structured questionnaire. Endobronchial ultrasound was performed using a CP-EBUS Olympus-BF-UC180F EBUS scope and the EU-ME2 ultrasound (Olympus). A 22-gauge Olympus needle was used for the EBUS-TBNA.

The bronchoscope was passed through the oral route. The endo-larynx, including vocal folds, tracheal lumen, carina, main bronchus, and bronchial segments were visualized. The usual standard was followed in the sampling procedure, which includes the highest lymph node station (N3), followed by N2 and then N1. Prior to the sampling procedure, the lymph node characteristics were assessed and noted.

To avoid the puncturing of vessels, Doppler mode was used to identify blood vessels in the lymph nodes and around the lymph nodes. Prior to the rapid onsite evaluation, each lymph node's EBUS characteristics were assessed and noted by two pulmonologists. A 22-gauge Olympus needle was used for sampling lymph nodes. After each pass, the sampled specimen was transferred to the pathology slides. Immediately, each slide was stained and evaluated for lymphocytes, granulomas, and atypical cells by an experienced pathologist. Each lymph node station was sampled up to three to four passes until onsite confirmation by a pathologist. The final pathology report was taken as a gold standard.

**Figure 1:**
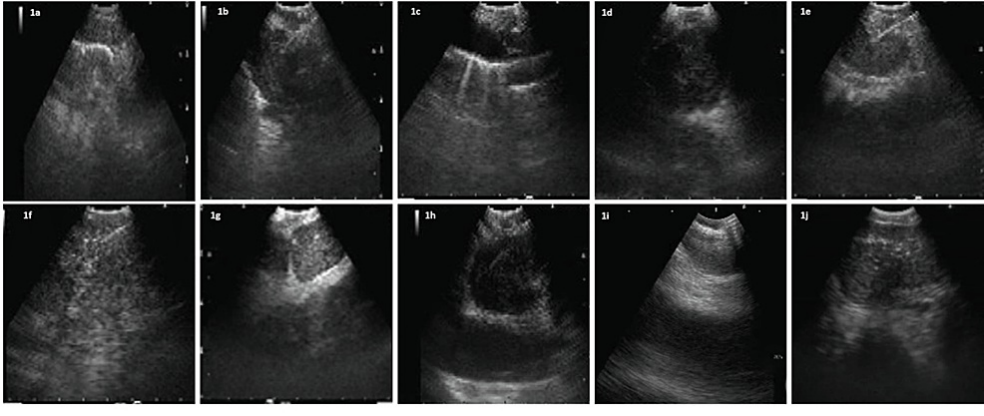
Representative images of endobronchial ultrasound images of lymphnodes. (a) Oval shape; (b) round shape; (c) small size (<10 mm); (d) large size (>10 mm); (e) distinct margins; (f) indistinct margins; (g) homogenous appearance; (h) heterogenous appearance; (i) central hilar structure absent; (j) coagulation necrosis sign present.

The following EBUS (ultrasonographic features) characteristics of lymph nodes were analyzed in our study. (i) Shape (oval vs round): the round shape was defined as a ratio of <1.5 between two perpendicular axes; (ii) margin (indistinct vs distinct): the distinct margin was defined as well-defined borders distinguished by a marked white line delimiting the LN; (iii) small axis <10 mm vs >10 mm; (iv) heterogeneous versus homogeneous echogenicity; (v) central hilar structure (central linear structure with high echogenicity): absent or present; (vi) coagulation necrosis (hypoechoic area within the lymph node without blood flow) sign: absent or present. These ultrasonographic features are compared with the final pathology outcome (benign or malignant) (Figure 1).

Statistical analysis

Statistical analysis was performed using IBM SPSS Statistics for Windows, Version 20.0 (Armonk, NY: IBM Corp.) analytic software. The categorical variables were expressed using frequency and percentage. To test the statistical significance of the association of categorical variables such as size, shape, echogenicity, central hilar structure, margins, and coagulation necrosis sign with the final diagnosis, Chi-square with continuity correction was used. Diagnostic measures such as sensitivity, specificity, predictive values, and accuracy of EBUS sonographic features were computed. A multivariate logistic regression analysis was performed to predict the most significant risk factors for malignant lymph nodes.

## Results

In the present study, a total of 576 patients were included; 366 were males, and the mean age was 54.9 ± 316.09 years. The inter-observer variability interclass coefficient was 0.9. Overall, 810 lymph nodes were assessed. Three hundred forty-eight cases (468 lymph nodes) were malignant, and 228 cases (342 lymph nodes) were benign. A total of 810 lymph nodes were assessed during EBUS and sampled. Among 810 lymph nodes, lymph nodes at station 7 were maximum (456) (Table 1).

**Table 1:** Comparative analysis of lymph nodal stations on endobronchial ultrasound in benign versus malignant.

Lymph node station*	Total	Malignancy	Benign
7	456	264	192
4R	210	138	72
4L	12	12	0
10R	30	18	12
10L	60	12	48
11L	36	30	6
2R	6	0	6

Among malignancies, in 348 patients, lung adenocarcinoma was most common (n=144), and among benign aetiology in 228 patients, tuberculosis was most common (n=96) (Table 2).

**Table 2:** The tabulation of final histopathological diagnosis of lymph node. *Excluding squamous cell carcinoma and adenocarcinoma of lung.

Final diagnosis	N= 576
Malignancy	
Adenocarcinoma of lung	144
Small cell carcinoma of lung	78
Squamous cell carcinoma of lung	60
Non-small cell carcinoma of lung*	24
Metastasis from distant sites	24
Poorly differentiated carcinoma	6
Primary T-cell lymphoma	6
Neuroendocrine carcinoma	6
Benign	
Tuberculosis	96
Sarcoidosis	84
Granulomatous lymph nodes	24
Nocardiosis	6
Mediastinal LN - adequate lymphocytes - improved with treatment - follow up showed no malignant conversion	6
Rt lower lobe non-resolving pneumonia with MLN - improved with antibiotics	6
Left-sided pleural effusion with MLN - follow up showed improvement	6

The comparison of EBUS nodal characteristics between benign and malignant diseases in the present study revealed the presence of heterogeneous echogenicity favoured malignant nodes over benign nodes. No significant correlation was found between the size, shape, margin, central hilar structure, coagulation necrosis sign, and final pathology report as benign or malignant (Table 3).

**Table 3:** Comparison of endobronchial ultrasound nodal characteristics between benign and malignant disease.

Nodal character	Malignant (n=468)	Benign (n=342)	P-value
Shape			
Oval	102 (70.8)	42 (29.2)	0.153
Round	366 (55)	300 (45)	
Size			
Small (<10 mm)	18 (37.5)	30 (62.5)	0.408
Large(>10 mm)	450 (59.1)	312 (40.9)	
Margin			
Distinct	306 (62.2)	186 (37.8)	0.196
Indistinct	162 (50.9)	156 (49.1)	
Echogenicity			
Homogeneous	30 (20)	120 (80)	<0.001
Heterogeneous	438 (66.4)	222 (33.6)	
Central hilar structure			
Preserved	24 (40)	36 (60)	0.395
Not preserved	444 (59.2)	306 (40.8)	
Coagulation necrosis sign			
Present	150 (50)	150 (50)	0.161
Not present	318 (62.4)	192 (37.6)	

Further, multivariate logistic regression analysis revealed that heterogeneous echogenicity was an independent predictor for malignancy (p-value < 0.001). Patients with heterogeneous echogenicity have a 7.8-times greater risk of having malignant lymph nodes (OR = 7.892, CI: 2.743-22.706).

The diagnostic yield of various EBUS ultrasonographic characteristics for malignant lymph nodes is represented in Table 4. The sensitivity, specificity, positive predictive value (PPV), negative predictive value (NPV), and diagnostic accuracy of heterogenous echogenicity for malignancy were 78.49%, 11.9%, 66.36%, 20%, and 57.78%, respectively.

**Table 4:** Diagnostic yield of various endobronchial ultrasound ultrasonographic characteristics for malignant lymph nodes. PPV: positive predictive value; NPV: negative predictive value.

Nodal character	Sensitivity	Specificity	PPV	NPV	Diagnostic accuracy
Round shape	78.21%	12.28%	54.95%	29.17%	50.37%
Large (>10 mm)	96.15%	8.77%	59.06%	62.50%	59.26%
Distinct margin	65.38%	45.61%	62.20%	49.06%	57.04%
Heterogeneous echogenicity	78.49%	11.90%	66.36%	20%	57.78%
Central hilar structure - not preserved	94.87%	10.53%	59.20%	60%	59.26%
Presence of coagulation necrosis sign	32.05%	56.14%	50%	37.65%	42.22%

## Discussion

With the evolution of EBUS, several studies have been conducted to evaluate ultrasound characteristics to predict the nature of the lymph node, whether malignant or benign.

Heterogeneous echogenicity on EBUS was initially observed in metastasis from medullary thyroid carcinoma owing to deposits of calcium and amyloid. The heterogenous appearance of lymph nodes can be attributed to a combination of hypoechoic (areas of liquefactive necrosis) and hyperechoic (areas of fibrosis and coagulation necrosis). Notably, normal, reactive, and tuberculous lymph nodes are hypoechoic when compared with adjacent muscles [1,2]. In the present study, conducted over a period of four years, for ultrasonographic features of mediastinal and hilar lymph nodes, heterogeneous echogenicity was the only statistically significant characteristic. These findings were consistent with previous studies by Schimid-Bindert et al. evaluating 281 lymph nodes. The study reported heterogeneous echogenicity as the single best lymph node characteristic to predict malignancy [3]. Similarly, Jhun et al. evaluated 172 lymph nodes, and on univariate analysis, lymph nodal characteristics predictive of metastasis were size greater than 10 mm, round shape, heterogeneous appearance, and absence of central hilar structure [7]. Fujiwara et al. showed that echogenicity, sensitivity and specificity, and diagnostic accuracy were 77.3%, 86.6%, and 83.9%, respectively, in 1061 lymph nodes, for heterogenous echogenicity [1]. The systematic review and metaanalysis comprising 29 studies of lymph nodal characteristics, reported an echogenicity pooled sensitivity of 0.61 [0.59-0.63], a pooled specificity of 0.82 [0.80-0.83], a diagnostic odds ratio of 6.04 [3.07-11.9], and a spearman’s correlation coefficient of 0.77 (SE = 0.04) 0.308 (p = 0.175). However, the meta-analysis found none of the EBUS features to be consistent with a diagnosis of malignant lymph node [10].

The size of lymph nodes is based on a small axis diameter; >10 mm is considered to be indicative of malignancy. The increase in the size of lymph nodes can be multifactorial, like tumor cell density and the proliferation of inflammatory cells. Even though malignant lymph nodes tend to be larger, in cases of micrometastases they can be small [11]. In cases where the ratio of the long-axis to short-axis diameter is less than 2 (i.e., a round shape), the lymph node is more likely to be malignant [12].

Fujiwara et al. reported that round shape, distinct margin, heterogeneous echogenicity, and presence of coagulation necrosis sign were independent predictive factors for metastasis [1]. Jessica et al. evaluated 227 lymph nodes in 100 patients with lung cancer and concluded that both the shape (round and oval) and the increasing size of lymph nodes are predictive of malignancy. However, no single feature could evidently discriminate between a malignant or benign node to avoid tissue diagnosis [4].

On the contrary, a study by Agarwal et al. observed significant differences between malignant and benign lymph nodes in terms of central hilar structure, margins, and coagulation necrosis sign, but not shape or size in differentiating benign and malignant lymph nodes [8]. The meta-analysis of 29 studies documented the presence of a round shape as a malignant lymph node has a pooled sensitivity of 81% and a pooled specificity of 65%. The present study also did not find size or shape to be predictive of benign or malignant etiology [10].

The malignant lymph node's sharp margins are attributable to the replacement of normal tissue by infiltration by tumor cells, leading to an increased difference in acoustic impedance. The irregular borders in benign etiology are due to the inflammation that causes peri adenitis. The margins as predictors have shown pooled sensitivity of 75% and specificity of 37% [10]. The present study documented a similar predictive value.

The CHS is formed by small lymph nodes and regular centrally located vascular structures; with further cellular proliferation, the CHS vanishes. This absence of CHS was reported to have a pooled sensitivity of 91% with an OR of 7.73, but with a pooled specificity of 37%, it may not be a good diagnostic test for malignancy [9,10]. The present study also documented a very high sensitivity but a very low specificity, indicating its inability to diagnose malignant lymph nodes.

The presence of coagulation necrosis signifies the absence of vascularity in the center as the tumor enlarges in size, leading to necrosis, known as the CNS [11]. Jhun et al. reported that the presence of coagulation necrosis was not a predictor of malignancy [7]. Similarly, Ayub et al. reported that the presence or absence of coagulation necrosis signs was not a predictor of benign disease [13]. The systematic review reported the CNS having the highest pooled specificity of 93% and an OR of 9.23 [95%CI 3.85-22.15] for the malignant lymph node [10]. However, the present study documented low diagnostic accuracy for CNS in predicting malignant lymph nodes.

The current study was a single-centre prospective observational study over a long period (four years) and included both benign and malignant cases of varied aetiology. Notably, ROSE (rapid on-site evaluation) for all the cases was done to increase the quality of the results. The current study had some limitations. First, the distribution of benign and malignant lymph nodes was unequal in the present study. Second, the gold standard used in the present study is a pathological diagnosis of transbronchial needle aspiration samples and not a surgical exploration of lymph nodes. Third, the interpretation of sonographic findings of targeted lymph nodes was performed in real-time during EBUS-TBNA for a relatively short period of time, and findings could be partly subjective according to investigators. Fourth, the ultrasonographic assessment will not preclude sampling of lymph nodes for final diagnosis. Lastly, the study did not compare the agreements and differences of CT or PET/CT scans to sonographic findings during EBUS.

## Conclusions

Lymph nodes with heterogeneous echogenicity have a higher probability of being malignant. The current study documented that lymph nodes with heterogeneous echogenicity have a 7.8 times higher probability of having malignancy. Also, the identification of lymph nodal characteristics on EBUS is helpful in decision-making in the background of non-diagnostic EBUS, whether to resample or not. When heterogenicity is observed on EBUS, subsequent sampling might be considered, which may yield a higher diagnostic yield and may reduce the number of lymph nodes requiring sampling and the need for further invasive procedures.
